# IRF2 is required for development and functional maturation of human NK cells

**DOI:** 10.3389/fimmu.2022.1038821

**Published:** 2022-12-05

**Authors:** Eva Persyn, Sigrid Wahlen, Laura Kiekens, Wouter Van Loocke, Hannah Siwe, Els Van Ammel, Zenzi De Vos, Filip Van Nieuwerburgh, Patrick Matthys, Tom Taghon, Bart Vandekerckhove, Pieter Van Vlierberghe, Georges Leclercq

**Affiliations:** ^1^ Laboratory of Experimental Immunology, Department of Diagnostic Sciences, Ghent University, Ghent, Belgium; ^2^ Cancer Research Institute Ghent (CRIG), Ghent, Belgium; ^3^ Department of Biomolecular Medicine, Ghent University, Ghent, Belgium; ^4^ Department of Pharmaceutics, Ghent University, Ghent, Belgium; ^5^ Laboratory of Immunobiology, Rega Institute for Medical Research, Department of Microbiology, Immunology and Transplantation, K.U. Leuven, Leuven, Belgium

**Keywords:** human NK cells, NK cell development, transcription factor, IRF2, NK cell biology

## Abstract

Natural killer (NK) cells are cytotoxic and cytokine-producing lymphocytes that play an important role in the first line of defense against malignant or virus-infected cells. A better understanding of the transcriptional regulation of human NK cell differentiation is crucial to improve the efficacy of NK cell-mediated immunotherapy for cancer treatment. Here, we studied the role of the transcription factor interferon regulatory factor (IRF) 2 in human NK cell differentiation by stable knockdown or overexpression in cord blood hematopoietic stem cells and investigated its effect on development and function of the NK cell progeny. IRF2 overexpression had limited effects in these processes, indicating that endogenous IRF2 expression levels are sufficient. However, IRF2 knockdown greatly reduced the cell numbers of all early differentiation stages, resulting in decimated NK cell numbers. This was not caused by increased apoptosis, but by decreased proliferation. Expression of IRF2 is also required for functional maturation of NK cells, as the remaining NK cells after silencing of IRF2 had a less mature phenotype and showed decreased cytotoxic potential, as well as a greatly reduced cytokine secretion. Thus, IRF2 plays an important role during development and functional maturation of human NK cells.

## Introduction

Natural killer (NK) cells are innate lymphoid cells that were first defined as cytotoxic effector cells that can kill target cells without prior sensitization. NK cells are also potent producers of inflammatory cytokines, such as interferon (IFN)-γ and tumor necrosis factor (TNF)-α (1). Like all lymphocytes, NK cells originate from hematopoietic stem cells (HSC) in the bone marrow, but terminal NK cell differentiation occurs in secondary lymphoid tissues (2, 3). Genetic regulation by an array of cell-intrinsic transcription factors and signaling events from cell-extrinsic factors, such as cytokines, direct the HSC development into NK cells. The commonly used model for human NK cell development is based on differential expression of the cell surface markers CD34, CD45RA, CD117, CD94 and CD16 to distinguish five successive NK cell developmental stages (4). CD34^+^ HSC that reside in the bone marrow develop into stage 1 cells once they acquire CD45RA, while retaining CD34 expression. Acquisition of CD117 marks stage 2 cells, which transition into stage 3 progenitor cells by downregulating CD34. Expression of CD122, the interleukin (IL)-2 receptor β chain, by stage 3 cells makes them responsive to IL-15 signaling and marks commitment to the NK cell lineage (5, 6). Acquisition of CD94 marks mature NK cells. Expression levels of CD56 and CD16 on mature NK cells divides them in CD56^bright^CD16^-^, i.e. stage 4 cells, which are potent cytokine producers, and CD56^dim^CD16^+^, i.e. stage 5 cells, which display mainly cytotoxic activity. Most peripheral blood NK cells are CD56^dim^, while the CD56^bright^ NK cells reside primarily in secondary lymphoid tissues (7). NK cell functional maturation is accompanied by the expression of NK cell receptors, like killer immunoglobulin-like receptors (KIR), NKG2A/C, NKp46, NKG2D and CD226 (DNAM-1) (7). Integration of signals received by the activating and inhibitory receptors expressed on the NK cell surface determines the outcome of NK cell activation.

The interferon regulatory factor (IRF) family contains nine transcriptional factors, from IRF1 to IRF9, in humans and mice. Members of the IRF family are involved in a variety of biological processes and also play diverse roles in immune cell development, differentiation and apoptosis (8). Several members of the IRF family are implicated in various aspects of NK cell biology, including differentiation and expansion. IRF8 is required for human NK cell development and functional maturation, as well as proliferative expansion after viral infection (9, 10). Two other members of the IRF family, IRF1 and IRF2, are known to be required for the development of murine NK cells. IRF1 regulates IL-15 expression in bone marrow stromal cells, which is essential for the development of NK cells. As a consequence, *Irf1*-knockout mice have strongly reduced NK cell numbers (11). IRF2 also affects murine NK cell development as *Irf2*-deficient mice show significantly decreased NK cell numbers, which arrest at the immature CD27^+^CD11b^–^ stage in the bone marrow (12–14). IRF2, in contrast to IRF1, acts in a cell-intrinsic manner as demonstrated by the fact that transplantation of *Irf2*-deficient bone marrow cells into irradiated wild-type recipient mice also results in decreased NK cell numbers as compared to transfer of control cells (12, 14). The selective loss of mature peripheral NK cells in *Irf2*-deficient mice is attributed at least partly to accelerated apoptosis, indicating a role for IRF2 in regulating NK cell survival, as well as maturation (14, 15).

To date, there are no reports on the role of IRF2 in human NK cell differentiation. Because of important interspecies differences, findings from mouse research cannot simply be extrapolated to the human situation and thus translational research is required. Here, using *in vitro* NK cell differentiation cultures starting from HSC that were transduced with IRF2 knockdown or IRF2 overexpression vectors, we show that the transcription factor IRF2 plays a critical role in human NK cell development. We uncovered that NK cell numbers are greatly reduced upon IRF2 knockdown and that this is due to a lower proliferation rate of the early developmental stages, whereas apoptosis is not affected. In addition, we show that the generated NK cells from the IRF2 knockdown cultures do not reach full NK cell functionality as they display impaired cytotoxicity against tumor target cells and reduced cytokine secretion upon cytokine stimulation. Overexpression of IRF2 has limited effects on NK cell maturation, indicating that endogenous IRF2 expression levels are sufficient in these processes.

## Material and methods

### Viral constructs

To knockdown the expression of the transcription factor, an IRF2-specific shRNA (5’-GCAATCCGGTGCCTTACAACA-3’) vector with a pLKO.1 backbone (Mission shRNA; Sigma Aldrich, St. Louis, MO, USA) was used. This lentiviral vector contained a puromycin resistance gene that was replaced by the enhanced green fluorescent protein (eGFP) reporter gene. After validation of the construct, viral supernatant was collected 48 h and 72 h after transfecting the lentiviral shRNA vectors together with pCMV-VSV-G envelope and p8.91 packaging vectors in HEK293T cells using JetPEI (Polyplus transfection, Illkirch, France). A non-targeting shRNA sequence was used as control.

To overexpress the transcription factor, IRF2 cDNA was cloned in the pCR-blunt vector using the Zero Blunt PCR Cloning kit (Thermo Fisher Scientific, Waltham, MA, USA), followed by subcloning into the LZRS-IRES-eGFP vector (16). After validation of the construct by sequencing, viral supernatant was collected 2, 6 and 14 days after transfecting the retroviral vectors in Phoenix A cells using calcium phosphate transfection. The empty LZRS-IRES-eGFP vector was used as control.

### Isolation of HSC

CD34^+^ cells were isolated from human umbilical cord blood (Cord Blood Bank, University Hospital Ghent, Ghent, Belgium). Cord blood was obtained with informed consent in accordance with the Declaration of Helsinki and usage was approved by the Ethics Committee of the Faculty of Medicine and Health Sciences (Ghent University, Ghent, Belgium). After isolation of mononuclear cells by Lymphoprep (Stem Cell Technologies, Grenoble, France) density gradient centrifugation, CD34^+^ cells were purified using Magnetic Activated Cell Sorting (MACS; Miltenyi Biotec, Leiden, The Netherlands). Isolated CD34^+^ cells were cultured in Iscove’s Modified Dulbecco’s Medium (IMDM; Thermo Fisher Scientific) containing fetal calf serum (FCS; Biowest, Nuaillé, France) (10%), penicillin (100 U/mL), streptomycin (100 µg/mL) and glutamine (2 mM) (all from Life Technologies, Grand Island, NY, USA), supplemented with thrombopoietin (TPO) (20 ng/mL), stem cell factor (SCF; Peprotech, London, UK) (100 ng/mL) and FMS-like tyrosine kinase 3 ligand (Ftl3L; R&D Systems, Minneapolis, MN, USA) (100 ng/mL). After 48 h of preculture, cells were transduced using RetroNectin (2 µg/cm²) (Takara Bio, Saint-Germain-en-Laye, France) coated plates. The addition of viral supernatant was followed by spinoculation at 950 g during 90 min at 32°C. In case of lentiviral transduction, polybrene (Sigma Aldrich) (8 µg/mL) was added during the transduction and 24 h after lentiviral transduction, the medium was refreshed to remove polybrene. eGFP^+^ HSCs, defined as CD34^+^lineage^-^(CD3/CD14/CD19/CD56)CD45RA^-^ cells, were sorted to high purity 48 h after transduction using a FACS ARIA II cell sorter (BD Biosciences, San Jose, CA, USA).

### Coculture model

The murine embryonic liver cell line EL08-1D2, kindly provided by E. Dzierzak (Erasmus University MC, Rotterdam, The Netherlands), was maintained on 0.1% gelatin-coated plates at 32°C in Myelocult M5300 medium (50%) (Stem Cell Technologies), α-MEM (35%), heat-inactivated FCS (15%), supplemented with penicillin (100 U/mL), streptomycin (100 µg/mL), glutamine (2 mM) and β-mercaptoethanol (10 μM). Cell proliferation was blocked by addition of mitomycin C (10 μg/mL) to the culture medium for 2-3 h, followed by thoroughly rinsing of the cells before harvesting using trypsin-EDTA (Lonza, Bazel, Switzerland). Cells were plated at a density of 50,000 cells per well of a 0.1% gelatin-coated tissue culture-treated 24-well plate at least 24 h before adding HSCs or differentiating NK cells.

Following FACS sorting, eGFP^+^ HSCs were plated on the mitomycin C-inactivated EL08-1D2. Cells were co-cultured in NK cell coculture medium containing Dulbecco’s Modified Eagle Medium (DMEM) and Ham’s F-12 nutrient mixture (2:1 ratio) (all from Thermo Fisher Scientific), supplemented with penicillin (100 U/mL), streptomycin (100 µg/mL), glutamine (2 mM), sodium pyruvate (10 mM) (Thermo Fisher Scientific), heat-inactivated human AB serum (20%) (Biowest), β-mercaptoethanol (24 µM), ascorbic acid (20 µg/mL), ethanolamine (50 µM) and sodium selenite (50 ng/mL) (all from Sigma Aldrich). The cytokines IL-3 (R&D systems) (5 ng/mL), IL-7 (20 ng/mL), IL-15 (10 ng/mL), SCF (20 ng/mL) and Ftl3L (10 ng/mL) were added to the culture medium. On day 7 of culture, the medium was refreshed by addition of equal volumes of fresh medium supplemented with cytokines (except IL-3). On day 14 of culture, the cells were split and transferred to new inactivated EL08-1D2 stromal cells. Cultures were maintained in a humidified atmosphere of 5% CO_2_ at 37°C.

### Flow cytometry analysis and sorting

Cells were harvested by forceful pipetting at indicated timepoints and immunostained for phenotypical analysis. *In vitro* NK developmental subsets were identified and analyzed using the following gating strategy on eGFP^+^ cells: HSC (CD34^+^CD45RA^-^), stage 1 (CD34^+^CD45RA^+^CD117^-^), stage 2 (CD34^+^CD45RA^+^CD117^+^), stage 3 (CD34^-^CD94^-^CD117^+^HLA-DR^-^NKp44^-^), stage 4 (CD45^+^CD56^+^CD94^+^CD16^-^) and stage 5 (CD45^+^CD56^+^CD94^+^CD16^+^) (**Supplemental Figure 1**).

To stain intracellular and intranuclear proteins, the BD Cytofix/Cytoperm (BD Bioscience) and Foxp3/Transcription Factor Staining Buffer Set (Thermo Fisher Scientific) were used, respectively.

Before staining, the cells were blocked with anti-mouse FcRgII/III (clone 2.4.G2) and human IgG (Miltenyi Biotec). To discriminate living and dead cells in cell membrane and intracellular or -nuclear staining, propidium iodide and Fixable Viability Dye eFluor™ 566 (Invitrogen) were used, respectively.

For apoptosis assays, cells were washed in annexin binding buffer and stained with annexin V-APC (Thermo Fisher Scientific) and propidium iodide.

Cells were analyzed on an LSRII (BD Biosciences); for sorting a FACSARIA was used. FlowJo_v10.8.1 (Ashland, OR, USA) was used for analysis. Utilized antibodies are listed in **Supplementary Table 1**.

### Cell proliferation assays

Cell proliferation was determined using the CellTrace™Violet Cell Proliferation kit (Thermo Fisher Scientific) following the manufacturer’s protocol and analyzed by flow cytometry at the indicated time point.

As a second method, the EdU assay was used. Coculture cells were labeled with 10 µM EdU (Click-iT EdU Alexa Fluor 594 Imaging Kit, Thermo Fisher Scientific) at 37°C, 5% CO_2_. After 30 min, the cells were harvested and stained extracellularly. Cells were then fixed in 3% paraformaldehyde for 20 min at room temperature. The cells were washed with PBS supplemented with 1% FCS and permeabilized for 10 min with ice-cold 0.2% Triton X-100 in PBS. Next, the cells were incubated 30 min at room temperature in the dark with 100 µL Click-iT reaction cocktail, prepared as instructed by the manufacturer. After washing with PBS, DNA was stained with DAPI (1 µg/mL) and cells were analysed by flow cytometry.

### Cytokine production and secretion

For flow cytometric analysis of cytokine production, coculture cells of day 21 were stimulated in bulk during 6 h with phorbol myristate acetate (PMA; 5 ng/mL) and ionomycin (1 µg/mL) or with K562 cells at an effector to target ratio (E:T) of 1:1, or during 24 h with IL-12 plus IL-18 (both 10 ng/mL) or IL-12, IL-18 and IL-15 (4 ng/mL). The last 4 h of incubation, brefeldin A (BD Golgiplug, BD Biosciences) was added. After harvesting, cells were stained for NK surface markers and subsequently fixed and permeabilized for intracellular staining of IFN-γ and TNF-α. For analysis of cytokine secretion, sorted mature eGFP^+^ NK cells (CD45^+^CD56^+^CD94^+^) from day 21 cultures were stimulated with IL-12 plus IL-18 or IL-12, IL-18 and IL-15 (same concentrations as indicated above). After 24 h, supernatant was collected and analyzed for cytokine secretion with IFN-γ ELISA assay (PeliKine-Tool Set, Sanquin, Amsterdam, The Netherlands) and TNF-α ELISA assay kits (TMB ELISA Development Kit, Peprotech).

### Cytotoxicity assay

K562 target cells (10^6^) were labeled with 100 µCi of ${\mathrm{Na}}_{2}^{\mathrm{51}}$ CrO_4_ (Perkin Elmer, Waltham, MA, USA) for 1 h at 37°C, 5% CO_2._ Labeled cells were washed three times in medium and resuspended in NK cell coculture medium. Cells were co-incubated with sorted eGFP^+^ NK cells at E:T ratios of 3, 1, 0.3, 0.1 and 0.03. Spontaneous release was measured by incubating target cells with medium alone, while maximum release was measured by incubating target cells in 1% Triton X-100. After 4 h, supernatant was harvested and mixed with scintillation fluid (Perkin Elmer). Radioactivity was measured with a 1450 LSC&Luminescence Counter (Wallac Microbeta Trilux, Perkin Elmer). The mean percentage of cytotoxic activity of triplicates was calculated.

### Western blot

Cells were lysed in RIPA buffer and protein concentration was determined using the DC protein assay (Bio-RAD, Hercules, CA, USA). Denatured protein was loaded on a Bolt 4-12% Bis-Tris Plus gel (Thermo Fisher Scientific) and transferred to a PVDF membrane (Invitrogen). After blocking, the membrane was incubated with the primary antibody at 4°C overnight, followed by incubation with the secondary antibody for 1 h. For visualization, anti-rabbit (#7074S, Cell Signaling Technologies) or anti-mouse (#NA931, Sigma Aldrich) conjugated horseradish peroxidase secondary antibody was used. Protein level quantification was performed using ImageJ software (National Institutes of Health). The primary antibodies used were: anti-IRF2 (#700226, Thermo Fisher; dilution 1:100) and anti-VINCULIN (#V9131, Sigma Aldrich; dilution 1:10000)

### qPCR analysis

Total RNA was extracted from sorted cells using the RNeasy Micro kit (Qiagen, Hilden, Germany) and converted into cDNA using the iScript™ Advanced cDNA synthesis Kit (Bio-RAD). Quantitative PCR was performed using the LightCycler 480 SYBR Green I Master mix (Roche, Bazel, Switzerland) on a LightCycler 480 real-time PCR system (Roche). The housekeeping genes GAPDH and either TBP or YHWAZ were used as normalization genes to calculate gene expression levels. Utilized primers are listed in **Supplementary Table 2**.

### Library preparation, RNA sequencing and analysis

For transcriptome analysis, day 3 HSC (eGFP^+^CD34^+^lineage^-^CD45RA^-^) and day 7 stage 3 cells (eGFP^+^CD45^+^CD34^-^CD117^+^CD94^-^NKp44^-^HLA-DR^-^) were sorted and RNA was isolated using the RNeasy Micro kit (Qiagen). The concentration and quality of the extracted RNA was checked using the ‘Quant-it ribogreen RNA assay’ (Life Technologies) and the RNA 6000 nano chip (Agilent Technologies, Santa Clara, CA, USA), respectively. The RNA sequencing libraries of 5 biological replicates of the HSC and stage 3 cells were prepared using the QuantSeq 3’ mRNA-Seq Library Prep Kit (Lexogen, Vienna, Austria) using 25 ng and 20.5 ng of input RNA, respectively. Libraries were quantified by qPCR, according to Illumina’s protocol ‘Sequencing Library qPCR Quantification protocol guide,’ version February 2011. A High Sensitivity DNA chip (Agilent Technologies) was used to control the library’s size distribution and quality. Sequencing was performed on a high throughput Illumina NextSeq 500 flow cell generating 75 bp single reads. Per sample, on average 3.8 x10^6^ ± 0.8 x 10^6^ and 4.4 x10^6^ ± 1.1 x10^6^ reads were generated for the HSC and stage 3 population, respectively. Quality control of these reads was performed with FastQC (17). Fastq files were aligned to human reference genome GRCh38 using STARv2.42 and gencode v35 as guide gtf. Counts were generated on the fly by STAR. Differential expression analysis was performed using Deseq2 with Wald test for p-value calculation (18). Genes with a padj < 0.05 were considered significantly differential. GSEA was performed using the GSEA software tool v4.2.1 of the Broad Institute (19, 20). The ‘GSEAPreranked’ module was run using standard parameters and 1000 permutations.

To test whether the differentially expressed genes (DEG) that are implicated in the gene ontology pathways of positive and negative regulation of the mitotic cell cycle contain an IRF2-binding motif, we used iRegulon. iRegulon is a computational method designed to reverse-engineer the transcriptional regulatory network underlying a co-expressed gene set using cis-regulatory sequence analysis. iRegulon implements a genome-wide ranking-and-recovery approach to detect enriched transcription factor motifs and their optimal sets of direct targets (21).

### Statistical analysis and software

Data were plotted and statistical analyses were performed using GraphPad Prism v8.3.1 software (GraphPad Software, San Diego, CA, USA). All error bars represent the standard error of the mean (SEM). Results were considered statistically significant when p < 0.05.

## Results

### IRF2 regulates the generation of human NK cells

**Figure 1:**
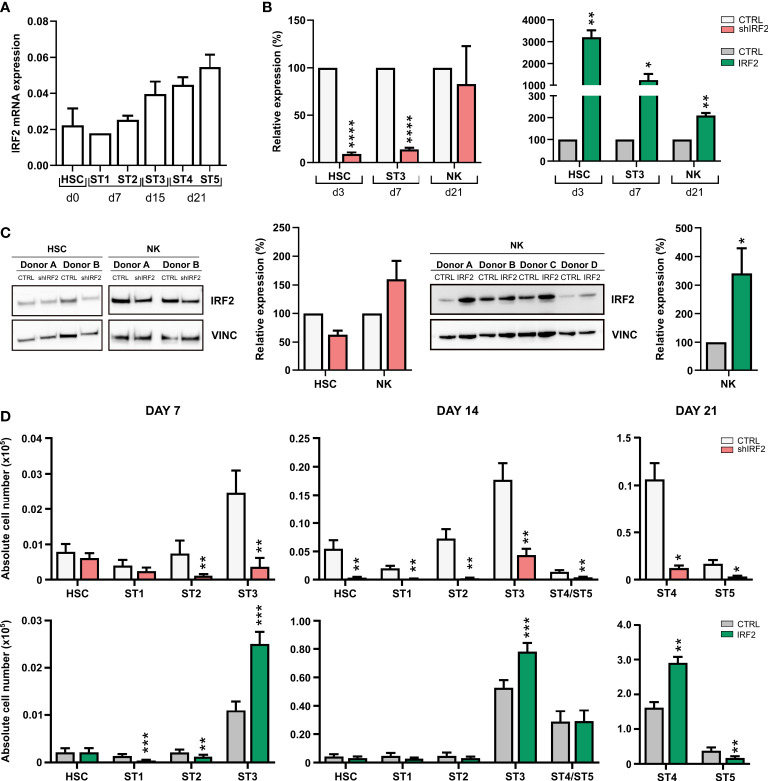
IRF2 regulates human NK cell differentiation. **(A)** RT-qPCR analysis of the IRF2 expression pattern in the indicated NK cell developmental stages sorted from HSC-based *in vitro* NK cell differentiation cultures: HSC (Lin^-^CD34^+^CD45RA^-^) sorted on day (d)0, stage (ST) 1 (CD34^+^CD45RA^+^CD117^-^) and ST2 (CD34^+^CD45RA^+^CD117^+^) on d7, ST3 (CD34^-^CD117^+^CD94^-^HLA-DR^-^NKp44^-^) on d15, and ST4 (CD56^+^CD94^+^CD16^-^) and ST5 (CD56^+^CD94^+^CD16^+^) on d21 (mean ± SEM; n=2-3). **(B)** Cord blood-derived HSC were transduced with IRF2 shRNA (left) or IRF2 overexpression vectors (right) and sorted eGFP^+^ HSC were cultured *in vitro* in NK cell specific culture conditions. Relative IRF2 expression was determined using RT-qPCR in sorted HSC at d3, in ST3 cells at d7 and in NK cells (CD45^+^CD56^+^CD94^+^) at d21. IRF2 expression is reported as mean percentage relative to the control condition (set at 100%) (mean ± SEM; n=3-5). **(C)** IRF2 Western blot analysis of sorted d3 HSC and d21 NK cells from knockdown (left) and d21 NK cells from overexpression (right) cultures. Bar graphs (right) show quantification of the IRF2 protein levels normalized to vinculin and reported relative to the control condition (set at 100%) (mean ± SEM; n=2-4). **(D)** Two days after transduction with IRF2 shRNA (top), IRF2 overexpression vector (bottom) or their appropriate controls, eGFP^+^ HSC were sorted and *in vitro* cultured in NK cell specific culture conditions. Absolute cell numbers of the successive NK cell developmental stages were determined at the indicated timepoints of the culture period (mean ± SEM; n=7-13). *, **, *** and **** represent statistical significance compared to control transduced cultures with p < 0.05, p < 0.01, p < 0.001 and p < 0.0001, respectively.

We first established the pattern of endogenous IRF2 expression during human NK cell development by performing RT-qPCR on cord blood-derived HSC and *in vitro* differentiation stages 1 to 5 (**Figure 1A**). IRF2 was clearly expressed in human HSC and stage 1 and 2 cells, and there was a gradual increase in IRF2 expression in the subsequent NK cell developmental subpopulations, i.e. stage 3 to stage 5.

To investigate the role of IRF2 in human NK cell development, we manipulated HSC, isolated from umbilical cord blood, to either knockdown or overexpress IRF2 by transducing them with a lentiviral vector containing an IRF2-specific shRNA or a retroviral vector containing IRF2 cDNA, respectively. As controls, a non-targeting shRNA or an empty IRES-eGFP control vector were used. HSC were sorted 2 days after transduction and put in NK cell differentiation culture on the EL08 stromal cell line. RT-qPCR analysis of subpopulations that were sorted at different timepoints of the IRF2 shRNA culture showed that HSC and stage 3 cells had significantly reduced IRF2 mRNA levels compared to control cells. However, the knockdown did not persist in the NK cell population. Retroviral transduction of IRF2 cDNA caused stable overexpression of IRF2 mRNA in HSC, stage 3 cells and NK cells (**Figure 1B**). Western blot analysis mirrored the mRNA levels, with a decrease in IRF2 protein expression in HSC, but not in NK cells upon IRF2 knockdown, whereas NK cells from overexpression cultures expressed significantly more IRF2 protein than control cell populations (**Figure 1C**).

To determine whether IRF2 plays a role in human NK cell development, cell numbers of the different NK cell differentiation stages were determined at weekly timepoints in IRF2 shRNA and IRF2 overexpression cultures by flow cytometric analysis (**Figure 1D**). On day 7, knockdown of IRF2 significantly reduced stage 2 and stage 3 cell numbers, while the numbers of HSC and stage 1 cells were unaffected. On day 14, however, also the numbers of HSC and stage 1 cells were significantly reduced compared to the control, and this was also the case for the emerging NK cells. This pattern continued on day 21, with strongly decreased stage 4 and stage 5 NK cell numbers. Conversely, overexpression of IRF2 resulted in increased stage 3 cell numbers on day 7 and day 14. This did not lead to more NK cells on day 14, but on day 21, an increase in stage 4 cell numbers was observed, whereas stage 5 NK cell numbers were decreased.

Taken together, endogenous IRF2 expression is upregulated during human NK cell maturation and IRF2 knockdown in HSC greatly reduces their differentiation into NK cells.

### IRF2 influences the transcriptome of HSC and stage 3 cells

**Figure 2:**
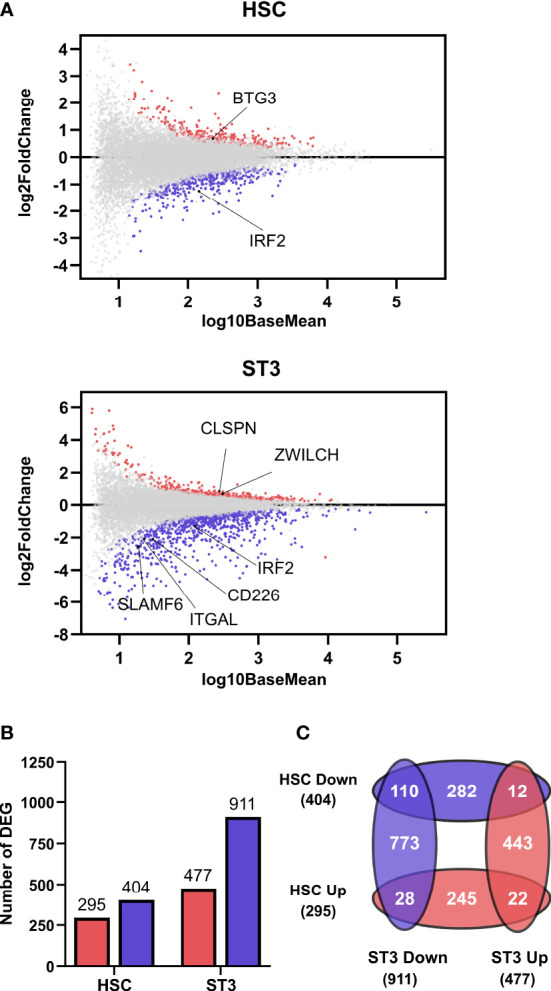
IRF2 knockdown affects the transcriptome of HSC and stage 3 cells. RNA-sequencing was performed on 5 biological replicates of HSC and ST3 cells sorted from IRF2 knockdown cultures on d3 and d7, respectively. **(A)** MA plots showing up- (red) and downregulated (blue) genres in HSC and ST3 cells. **(B)** Bar graphs showing the number of differentially expressed genes (DEG) in HSC and ST3 that were up- (red) or downregulated (blue). **(C)** Venn diagram showing the overlap between the DEGs in the HSC and ST3 populations.

The decreased cell numbers of the early NK cell differentiation stages indicated towards an early effect of IRF2 on NK cell development. To investigate how IRF2 knockdown might influence NK cell development, we performed transcriptome analysis on day 3 HSC and day 7 stage 3 cells sorted from the knockdown and control cultures. Consistent with the reported ability of IRF2 to act as both a transcriptional activator and repressor (22), 295 transcripts were upregulated and 404 transcripts were downregulated in the HSC population, while in the stage 3 cells 477 transcripts were upregulated and 911 transcripts were downregulated in the IRF2 knockdown compared to the control condition (**Figure 2A, B**). Comparative analysis of the DEG of the HSC and stage 3 populations showed 22 and 110 genes that were up- and downregulated, respectively, in both populations, whereas 40 genes in total showed opposing differential expression in the two populations (**Figure 2C**).

### IRF2 affects proliferation of early differentiation stages

**Figure 3:**
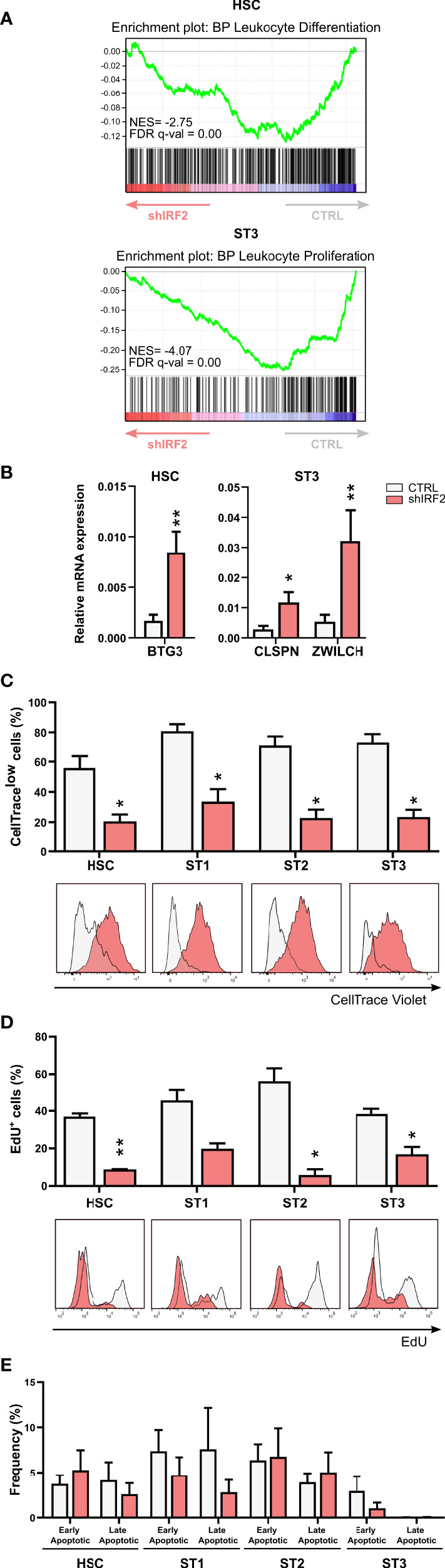
IRF2 influences proliferation of early NK cell developmental stages. **(A)** Gene Set Enrichment Analysis plot of the Gene Ontology – Biological Process (GO-BP) leukocyte differentiation and leukocyte proliferation pathway upon IRF2 knockdown in HSC and ST3 cells, respectively. NES: normalized enrichment score. **(B)** Relative expression of BTG3 in HSC and CLSPN and ZWILCH in ST3 cells as analyzed by RT-qPCR (mean ± SEM; n=4). **(C-E)** HSC were transduced with IRF2 knockdown or control vectors. **(C)** eGFP^+^ HSC, sorted after transduction (d0), were labeled with CellTrace Violet and cultured in NK cell specific conditions. On d5, the CellTrace Violet signal was assessed in gated HSC and ST1 to ST3 cells with flow cytometry. The frequency of CellTrace^low^ cells is indicated (mean ± SEM; n=5). Overlaid CellTrace Violet histograms of representative samples are shown. **(D)** eGFP^+^ HSC were cultured for 5 days. Thereafter, cells were incubated with 5-ethynyl-2’-deoxyuridine (EdU) during 30 min, followed by a Click-iT reaction with an Alexa Fluor 594 fluorophore and flow cytometric quantification. The percentages of EdU-incorporating cells in the indicated stages are shown (mean ± SEM; n=3). **(E)** Apoptosis was assessed on d5 of culture by flow cytometry in the indicated developmental stages by staining with propidium iodide (PI) and Annexin V. The percentage of early (Annexin V^+^PI^-^) and late apoptotic cells (Annexin V^+^PI^+^) is shown (mean ± SEM; n=5). * and ** represent statistical significance compared to control transduced conditions with p < 0.05 and p < 0.01, respectively.

We performed Gene Set Enrichment Analysis (GSEA) of the RNA-seq datasets and this showed that leukocyte differentiation and leukocyte proliferation were among the suppressed biological processes in the HSC and stage 3 cells from the IRF2 knockdown cultures, respectively (**Figure 3A**). Further analysis of the DEGs revealed that several genes involved in negative regulation of mitotic cell cycle, such as BTG3 in the HSC population and ZWILCH and CLSPN in stage 3, were upregulated in IRF2 knockdown cells. BTG3 is a member of the antiproliferative BTG gene family, and its downregulation has been observed in human cancers (23). ZWILCH, an essential component of the mitotic checkpoint, prevents cells from prematurely exiting mitosis and is involved in the negative regulation of mitotic cell cycle (24). Claspin (encoded by the CLSPN gene) is a nuclear protein recognized to regulate cell cycle S-phase checkpoint (25). RT-qPCR analysis of sorted cell populations from IRF2 knockdown cultures revealed higher expression of BTG3 in HSC and of ZWILCH and CLSPN in stage 3 cells compared to control cultures (**Figure 3B**), confirming the RNA-seq results. There were, however, both up- and downregulated DEGs among the gene ontology pathway of the positive regulation, as well as the negative regulation of the mitotic cell cycle. We used iRegulon to identify the DEGs implicated in the gene ontology pathway of positive or negative regulation of the mitotic cell cycle that contain an IRF2-binding motif (**Table S3**, with genes containing an IRF2 binding motif in bold). Therefore, we analyzed the proliferation rate in the knockdown culture using two different experimental approaches. In the first approach, sorted eGFP^+^ HSC were labeled on day 0 with CellTrace Violet and were put in NK cell differentiation culture. CellTrace labels the cells fluorescently and upon cell division the daughter cells receive approximately half of the fluorescent label of the parent cells. On day 5, the CellTrace signal was analyzed in the gated early NK cell stages. The results show that all differentiation stages, i.e. HSC and stages 1 to 3, from IRF2 knockdown cultures had a significantly decreased percentage of CellTrace^low^ cells (**Figure 3C**). In the second approach, we labeled cells from day 5 cultures with 5-ethynyl-2’-deoxyuridine (EdU). EdU is a modified thymidine analogue that is efficiently incorporated into newly synthesized DNA. Afterwards, it is labeled with a bright fluorescent dye in a highly-specific click reaction. All differentiation stages from the IRF2 knockdown cultures had a significant decreased uptake of EdU and thus contain less cells entering the S-phase of the cell cycle (**Figure 3D**). This confirms and strengthens the results of the CellTrace experiments and shows that proliferation of the early differentiation stages is strongly decreased upon IRF2 knockdown.

As also apoptosis affects NK cell numbers, we stained the cells from the IRF2 knockdown and control cultures with annexin V and propidium iodide on day 5. This showed that there was no significant difference in the frequency of apoptotic cells (**Figure 3E**).

Thus, IRF2 knockdown strongly decreases proliferation of the early developmental stages, whereas it does not affect apoptosis.

### IRF2 is required for human NK cell cytotoxicity and cytokine secretion

**Figure 4:**
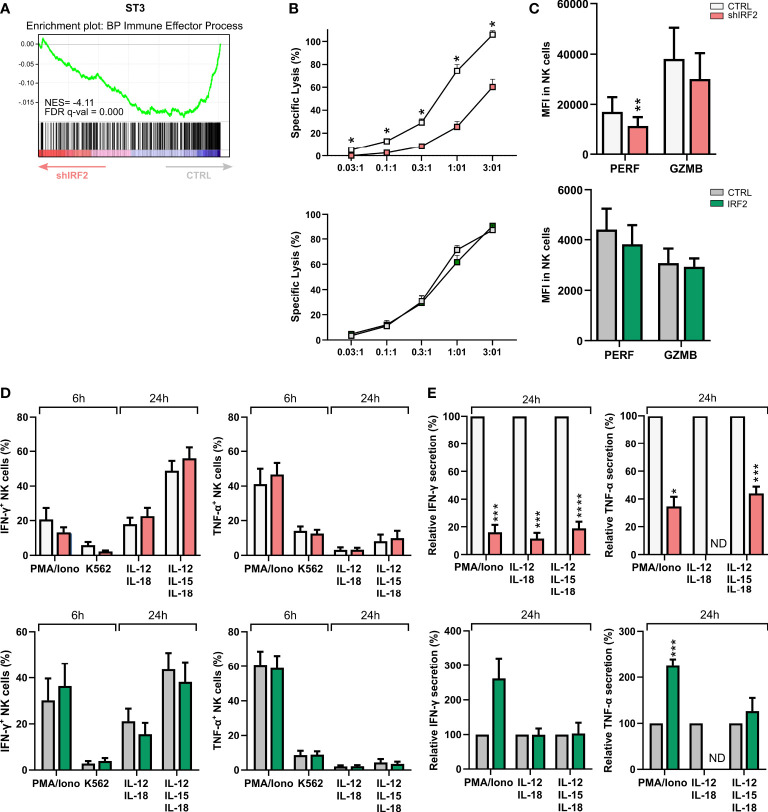
Decreased cytotoxicity and cytokine secretion upon IRF2 knockdown. **(A)** Gene Set Enrichment Analysis plot of the GO-BP immune effector process upon IRF2 knockdown in ST3 cells. NES: normalized enrichment score. **(B-E)** Functionality of NK cells of IRF2 knockdown and overexpression conditions was examined on d21 of culture. **(B)** NK cell cytotoxicity was assessed using a 51-chromium release assay. NK cells (eGFP^+^CD45^+^CD56^+^CD94^+^) were sorted and incubated for 4 h with K562 target cells at the indicated effector to target (E:T) ratio. The percentage of specific target cell lysis is shown (mean ± SEM; n=3-7). **(C)** Expression (MFI) of the cytotoxic mediators perforin (PERF) and granzyme B (GZMB) in gated NK cells (mean ± SEM; n=8-10). **(D)** IFN-γ and TNF-α production was analyzed with flow cytometry in gated NK cells after stimulation of bulk cells with PMA/Ionomycin or by coculture with K562 target cells for 6 h, or after 24 h stimulation with either IL-12 plus IL-18 or IL-12, IL-15 and IL-18. Brefeldin A was added during the last 4 h of stimulation (mean ± SEM; n=6-10). **(E)** NK cells were sorted and stimulated with IL-12 plus IL-18 or IL-12, IL-15 and IL-18. After 24 h, the supernatant was harvested and IFN-γ and TNF-α secretion was analyzed by ELISA. Cytokine secretion is reported as mean percentage relative to the control condition (set at 100%) (mean ± SEM; n=4-6). ND: not detectable. *, **, *** and **** represent statistical significance compared to control transduced cultures with p < 0.05, p < 0.01, p < 0.001 and p < 0.0001, respectively.

NK cells are important cytotoxic players of the innate immune system. GSEA of the RNA-seq data also revealed immune effector process among the pathways that were significantly decreased in stage 3 cells of the IRF2 knockdown condition (**Figure 4A**). This compelled us to analyze the effector functions of the mature NK cells generated upon altered IRF2 expression. First, we assessed if IRF2 expression is necessary for NK cell cytotoxic function. We performed chromium release assays using the NK-sensitive K562 cancer cell line as target cells. Knockdown of IRF2 significantly impaired tumor killing of sorted NK cells at all examined effector to target ratios, while cells of overexpression cultures had a similar cytotoxic capacity as control NK cells (**Figure 4B**). Perforin and granzyme B are key effector molecules of NK cell cytotoxicity. Using intracellular staining, we found a limited but significant decrease in perforin expression in NK cells upon knockdown of IRF2, while granzyme B expression showed a non-significant trend of decreased expression. Overexpression of IRF2 had no influence on perforin or granzyme B expression levels (**Figure 4C**).

Mature NK cells are also characterized by their ability to produce and secrete pro-inflammatory cytokines, such as IFN-γ and TNF-α. Cells of knockdown and overexpression cultures were stimulated for 6 h with PMA/ionomycin or with K562 target cells, or for 24 h with IL-12 plus IL-18, with or without IL-15. Flow cytometric analysis of the frequency of NK cells producing IFN-γ or TNF-α showed no difference in knockdown or overexpression cultures compared to their respective control (**Figure 4D**). In sharp contrast to cytokine production, cytokine secretion was strongly affected by IRF2 knockdown. This was assessed with IFN-γ and TNF-α ELISA after 24 h stimulation with either PMA/ionomycin or the cytokines mentioned earlier. Knockdown of IRF2 strongly decreased secretion of both IFN-γ and TNF-α. IRF2 overexpression only had limited influence, with an increase in secreted TNF-α upon PMA/ionomycin stimulation (**Figure 4E**).

These results show that differentiating human NK cells require IRF2 to acquire full tumor cytotoxicity and cytokine secretion capacities.

### IRF2 is required for full phenotypic maturation of human NK cells

**Figure 5:**
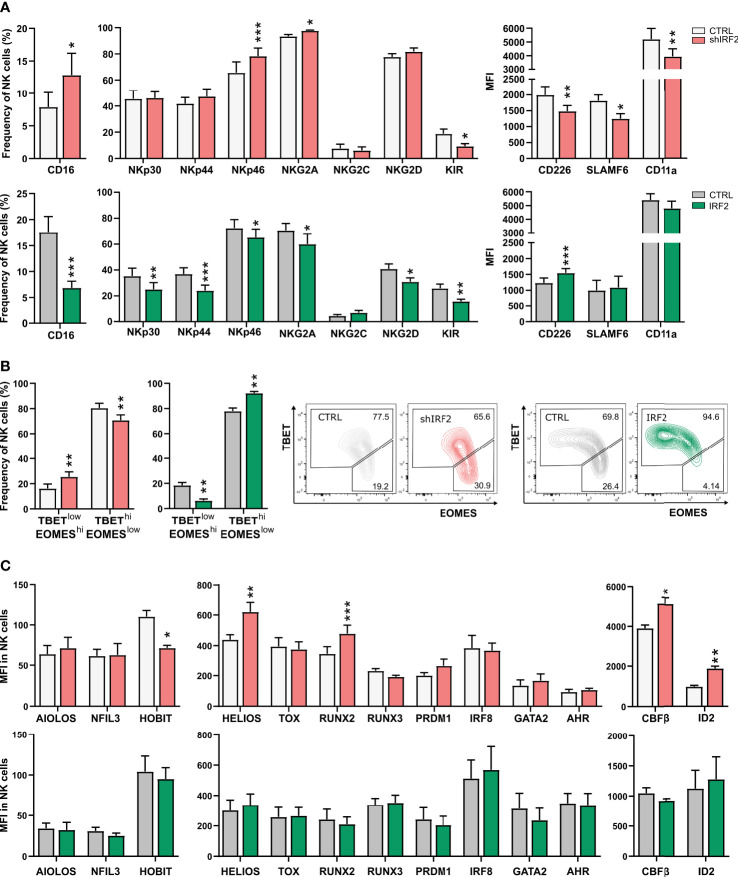
IRF2 is required for NK cell functional maturation. **(A)** On d21 of culture, NK cells (eGFP^+^CD45^+^CD56^+^CD94^+^) of IRF2 knockdown (top) or overexpression (bottom) conditions were analyzed for expression of the indicated cell membrane NK cell markers by flow cytometry (mean ± SEM; n=8-14). **(B)** Percentage of NK cells with a TBET^hi^EOMES^low^ or TBET^low^EOMES^hi^ phenotype on d21 of IRF2 knockdown (left) and overexpression (right) cultures (mean ± SEM; n=9-10). Representative dot plots are shown. **(C)** The expression (MFI) of the indicated transcription factors was determined by flow cytometry on d21 in NK cells of IRF2 knockdown (top) and overexpression (bottom) cultures (mean ± SEM; n=4-12). *, ** and *** represent statistical significance compared to control transduced cultures with p < 0.05, p < 0.01 and p < 0.001, respectively.

Recognition of target cells by NK cells is mediated by an array of activating and inhibitory receptors expressed on their cell surface. Evaluation of multiple NK cell receptors on NK cells from day 21 of IRF2 knockdown and overexpression cultures showed that expression of both activating and inhibitory receptors was affected (**Figure 5A**). The Fc gamma receptor CD16, which marks stage 5 cells, was upregulated in NK cells of IRF2 knockdown and downregulated in overexpression cultures. Like CD16, the activating receptor NKp46 and inhibitory receptor NKG2A were upregulated upon IRF2 knockdown, and downregulated when IRF2 was overexpressed. KIR receptors, stained with a mix of KIR antibodies (KIR2DL1/KIR2DS1, KIR2DL2/KIR2DL3, KIR3DL1/KIR3DS1) labeling both activating and inhibitory KIRs, showed decreased expression frequency in both knockdown and overexpression cultures. Additionally, NKp30, NKp44 and NKG2D were downregulated in IRF2 overexpression cultures, but unaltered in the knockdown cultures. Expression of the co-receptors CD226 (DNAM-1) and SLAMF6 was downregulated in IRF2 knockdown cultures, with CD226 also upregulated in overexpression cultures. NK cells also express adhesion molecules on their cell surface besides the activating and inhibitory receptors. Strong adhesion to target cells is a requirement for efficient killing by NK cells. CD11a, the alpha chain of the integrin LFA-1, showed decreased expression levels in NK cells from knockdown cultures, while expression levels were not altered in overexpression cultures. Notably, the RNA-seq analysis revealed that expression of *CD226*, *SLAMF6* and *ITGAL* (encodes CD11a) were already downregulated in stage 3 cells of the IRF2 knockdown cultures, contributing to the downregulation of the immune effector pathway in this population.

TBET and EOMES are two crucial transcription factors in murine and human NK cell differentiation and activation. During NK cell maturation, TBET is upregulated, while EOMES is downregulated (26, 27). NK cells thus evolve from an immature TBET^low^EOMES^hi^ to a mature TBET^hi^EOMES^low^ phenotype. Comparison of the TBET and EOMES expression profile in the NK cell population of IRF2 knockdown versus control cultures revealed a shift to the immature phenotype, whereas overexpression cultures showed the opposing expression pattern (**Figure 5B**).

We also assessed the expression of a large panel of transcription factors important in NK cell development or function upon altered IRF2 expression. There was increased expression of HELIOS, RUNX2, CBFβ and ID2 and decreased expression of HOBIT in NK cells from IRF2 knockdown cultures. Overexpression of IRF2 did not alter the expression of any of the examined transcription factors (**Figure 5C**).

Thus, NK cells from IRF2 knockdown cultures do not express a full mature phenotype of cell membrane receptors and transcription factors.

## Discussion

In the past decade, NK cell based therapies have rapidly emerged in the field of cellular therapy as a promising approach to treat cancer. Although transfusion of NK cells has demonstrated safety, both in autologous and allogeneic settings, challenges still remain to achieve sufficient clinical efficacy (28, 29). Strategies for the development of NK cell based therapies currently focus on the source from which NK cells are derived and on enhancing NK cell potency and persistence (30). Several sources of NK cells are being investigated, including peripheral blood NK cells, NK cell lines and stem cell-derived NK cells (31). Stem cell-derived NK cells offer a promising resource as they provide the ability to manipulate the differentiation process and generate a standardized off-the-shelf therapy (31). In order to achieve the required numbers of NK cells with optimal immunoregulatory and cytotoxic functions, a more complete understanding of human NK cell differentiation and maturation is needed. In recent years, extensive research on NK cell development led to the identification of different developmental stages and several transcription factors essential in the process (6). However, most of our knowledge is derived from genetically modified mice models and the knowledge on human NK cells remains limited.

We examined the endogenous expression of IRF2 in successive NK cell developmental stages and found that IRF2 expression levels in stage 1 and stage 2 cells were similar as in the HSC population, and expression was upregulated during NK cell differentiation from stage 3 cells onwards with the highest expression level in stage 5 NK cells. This expression pattern suggests that IRF2 plays a role in human NK cell development. *Irf2*-deficient mice display greatly reduced numbers of NK cells, with a marked decrease of mature NK cells in the periphery, while NK cell numbers in the bone marrow are less affected (12, 14). We investigated how IRF2 regulates human NK cell differentiation and function by creating *in vitro* NK cell differentiation cultures starting with cord blood-derived HSC that were transduced with IRF2 knockdown or IRF2 overexpression vectors. We showed that altered expression of IRF2 greatly influences the absolute cell numbers of not only NK cells but also of their early progenitor stages. Most notably, stage 3 cell numbers were decreased in IRF2 knockdown and increased in IRF2 overexpression cultures, ultimately leading to a subsequent decrease and increase of stage 4 NK cells, respectively.

Transcriptome analysis of HSC and stage 3 cells of IRF2 knockdown cultures revealed that leukocyte differentiation and leukocyte proliferation pathways were downregulated in the HSC and stage 3 knockdown cells, respectively. Together with the compromised cell numbers that we observed from day 7 in IRF2 knockdown cultures, this prompted us to asses proliferation and apoptosis in the early NK cell differentiation stages upon IRF2 knockdown in HSC. In contrast to the mouse context, where the reduced NK cell number in *Irf2*-deficient mice, especially in the periphery, could at least partly be attributed to an increased apoptotic rate (14, 15), the decreased cell numbers in human IRF2 knockdown cultures were not due to increased apoptosis. In contrast, CellTrace and EdU-labeling experiments showed that all early differentiation stages, including HSC and stage 1 to stage 3 cells, had a much lower proliferation rate upon IRF2 knockdown. These results are in agreement with published papers describing a positive regulation of the cell cycle by IRF2. In embryonic fibroblasts, IRF2 stimulates proliferation by regulating the transcription of histone H4 (32) and knockdown of IRF2 also inhibits cell proliferation in many leukemic cell lines (33–35). However, an opposing role for IRF2 has also been described as the knockout of IRF2 in human primary keratinocytes increases self-renewal (36) and mice deficient in IRF2 show increased basophil expansion (37).

Knockdown of IRF2 in HSC did not persist until day 21 of culture, with similar expression levels of IRF2 in NK cells as in the control culture. It was therefore unexpected that NK cells generated from progenitors with decreased IRF2 expression did display impaired functional maturation. Indeed, NK cells from IRF2 knockdown cultures demonstrated impaired cytokine secretion and defective cytotoxicity towards NK-sensitive K562 target cells. This indicates a role for IRF2 in the acquisition of effector activity, whereas it is not required for its maintenance. A similar phenomenon has been observed with other transcription factors, e.g. NFIL3 and ETS1. Indeed, human HSC transduced with a dominant-negative isoform of ETS1 generate NK cells that show defective cytotoxicity and higher IFN-γ secretion, while transduction of mature NK cells with this dominant-negative variant does not affect NK cell functionality (38). *Nfil3*-deficient mice have severely reduced NK cell numbers, and the few NK cells that do develop are poorly cytotoxic and produce less IFN-γ (39). However, conditional deletion of *Nfil3* during the immature NK cell stage has no effect on NK cell development or cytokine production (40).

In contrast to NK cells from *Irf2*-deficient mice that exhibit similar cytotoxic killing as those from control mice (12), human NK cells from the IRF2 knockdown cultures showed reduced cytotoxicity. NK cell cytotoxicity is a complex process, in which several sequential steps ultimately result in degranulation and the release of cytotoxic effector molecules that kill the target cell. These include NK cell adhesion to potential target cells, formation of an immunological synapse, NK cell activation, and translocation of cytotoxic granules to the immunological synapse (41). A defect in any of these steps reduces NK cell cytotoxicity. NK cells from IRF2 knockdown cultures showed decreased expression of CD11a, together with decreased CD226 and SLAMF6 expression. Expression of CD11a (the α-chain of LFA-1) is crucial in adhesion of NK cells to potential target cells. Upon recognition of a target cell, an immunological synapse is formed between the NK cell and the target cell, in which LFA-1 plays an essential role (42). NK cells from LFA-1-deficient mice are unable to kill target cells (43), and antibody blocking of LFA-1 impairs human NK cell cytotoxicity due to impaired conjugate formation (44). Binding of CD226 to its ligands CD112 and CD155, which are abundantly expressed by K562 cells, is known to promote NK cell cytotoxicity and IFN-γ production (45, 46). Interestingly, CD226-mediated NK cell activation is dependent on its association with LFA-1, as demonstrated by the impaired CD226-mediated cytotoxicity of NK cells from patients with leukocyte adhesion deficiency syndrome, who have a deficiency of the LFA-1 beta subunit (47). Additionally, SLAMF6 triggering is sufficient to induce activation of LFA-1 (42). Homophilic interaction of SLAMF6 promotes the cytolytic activity of NK cells and influences IFN-γ production (48). Furthermore, perforin expression is decreased in the NK cells from IRF2 knockdown culture, which also contributes to the defective cytotoxicity of IRF2 knockdown NK cells. In this regard, our finding that TBET expression is lower in NK cells from IRF2 knockdown cultures is important, as the role of TBET in regulating NK cell cytotoxicity through expression of perforin and granzyme B is well established (49).

Alongside their direct cytotoxicity, activated NK cells also release pro-inflammatory cytokines. While *Irf2*-deficient mice displayed less IFN-γ-producing NK cells, mainly in the CD11b^low^ fraction (12), the frequencies of IFN-γ- or TNF-α-producing NK cells were not altered in IRF2 knockdown cultures, whereas the NK cells did release significantly less cytokines. While NK cells produce IFN-γ in greater abundance than TNF-α, both cytokines are trafficked and released simultaneously (50). As the release of both IFN-γ and TNF-α are similarly affected, this suggests a defect in cytokine secretion. Opposed to the release of cytotoxic granules, little is known about how cytokines are secreted by NK cells (50). We hypothesize that knockdown of IRF2 affects expression of a protein that directly regulates this secretion process. Interestingly, NK cells from IRF2 knockdown cultures showed increased expression of RUNX2, in addition to elevated CBFβ expression. All RUNX transcription factors dimerize with CBFβ, which enhances their DNA binding affinity (51). Recently, the role of RUNX2 in human NK cell biology was uncovered, and this revealed that RUNX2 negatively impacts the production and secretion of IFN-γ (52). However, the exact mechanism how cytokine secretion is affected in NK cells from IRF2 knockdown cultures remains elusive at this time.

Besides RUNX2 and CBFβ, NK cells from the knockdown cultures also exhibited upregulated expression of other transcription factors important in NK cell development and/or function, including ID2 and HELIOS. Upregulated ID2 does not correspond to decreased NK cell numbers in IRF2 knockdown cultures as, at least in mouse, ID2 deficiency arrests NK cell maturation at the immature CD27^+^CD11b^-^ stage (53, 54). However, the upregulated HELIOS expression is consistent with the fact that NK cells from IRF2 knockdown cultures do not achieve a full mature phenotype, as it has been shown that HELIOS is predominantly expressed in the earliest stages of NK cell maturation, and downregulated in the mature NK cell population (55). Maturation of NK cells is also accompanied by upregulation of TBET and downregulation of EOMES (27), and we showed that the TBET and EOMES expression profile in IRF2 knockdown cultures shifts toward a more immature phenotype. Finally, HOBIT, that is downregulated in IRF2 knockdown cultures, has been shown to be important in human NK cell development as knockdown of HOBIT in cord blood cells results in decreased generation of NK progenitors and CD56^+^ NK cells (56).

In conclusion, our results show that IRF2 regulates the generation of human NK cells and that its expression is required during functional maturation of NK cells so that they acquire their full cytotoxic and cytokine secretion potential.

## Data availability statement

The datasets presented in the study are accessible in the GEO repository, accession number GSE212023.

## Ethics statement

The usage of human umbilical cord blood in this study was reviewed and approved by the Ethics Committee of the Faculty of Medicine and Health Sciences, Ghent University, Ghent, Belgium. The patients/participants provided their written informed consent to participate in this study.

## Author contributions

Conceptualization, E.P. and G.L.; methodology, E.P. and G.L.; investigation, E.P., S.W., L.K., H.S., Z.D.V., F.N. and E.V.A.; resources, P.M., T.T., B.V., P.V.V. and G.L.; software, W.V.L.; supervision, G.L.; writing –original draft preparation, E.P.; writing –review and editing, E.P. and G.L.; visualization, E.P.; funding acquisition, G.L., L.K., S.W. All authors have read and agreed to the submitted version of the manuscript.

## Funding

This research was supported by grants from the Research Foundation – Flanders (FWO), including G.0444.17N (G.L.), 1S29317N (L.K.), 1S45317N (S.W.).

## Acknowledgments

Practical expertise and assistance regarding RNA-seq used in this study was provided by NXTGNT, Ghent, Belgium. The computational resources (Stevin Supercomputer Infrastructure) and services used in this work were provided by the VSC (Flemish Supercomputer Center), funded by Ghent University, FWO and the Flemish Government – department EWI.

## Conflict of interest

The authors declare that the research was conducted in the absence of any commercial or financial relationships that could be construed as a potential conflict of interest.

## Publisher’s note

All claims expressed in this article are solely those of the authors and do not necessarily represent those of their affiliated organizations, or those of the publisher, the editors and the reviewers. Any product that may be evaluated in this article, or claim that may be made by its manufacturer, is not guaranteed or endorsed by the publisher.
