# Supplementary material for: IRF2 is required for development and functional maturation of human NK cells
Source: Front Immunol. 2022 Dec 5;13:1038821. doi: 10.3389/fimmu.2022.1038821 (PMC9762550; doi:10.3389/fimmu.2022.1038821)
Supplement: Supplementary file 1 [file DataSheet_1.pdf]

Supplementary Material

1 Supplementary Table 1. Utilized antibodies for flow cytometry

Marker	Alternative name	Fluorochrome	Clone	Supplier
**CD3**	CD3	APC	SK7	BD Biosciences
**CD11a**	LFA-1	PE, Alexa Fluor 700	HI111	Biolegend
**CD14**	CD14	APC	REA599	Miltenyi Biotec
**CD16**	FcγRIIIA	PE, APC	B73.1	Biolegend
**CD19**	CD19	APC	SJ25C1	Thermo Fisher Scientific
**CD34**	CD34	PE, PB	AC136	Miltenyi Biotec
**CD45**	CD45	APC/Fire™750 (APC-Fire)	2D1	Biolegend
**CD56**	NCAM1	VioBlue (VB), APC	5.1H11	Biolegend
**CD94**	KLRD1	PerCP-Cy5.5	DX22	Biolegend
**CD117**	KIT	PECy7	104D2	Thermo Fisher Scientific
**CD158a,h**	KIR2DL1/KIR2DS1	PE	REA1010	Miltenyi Biotec
**CD158b1/b2**	KIR2DL2/KIR2DL3	PE	REA1006	Miltenyi Biotec
**CD158e1/e2**	KIR3DL1/KIR3DS1	PE	REA168	Miltenyi Biotec
**CD158i**	KIR4DS1	PE	REA860	Miltenyi Biotec
**NKG2A**	CD159a	APC	REA110	Miltenyi Biotec
**NKG2C**	CD159c	PE	FAB138P	R&D systems
**CD226**	DNAM-1	Alexa Fluor 647	DX11	BD Biosciences
**NKG2D**	CD314	APC	1D111	Biolegend
**NKp46**	CD335;NCR1	PECy7	9E2	Biolegend
**NKp44**	CD336;NCR2	eFluor450, APC	44.189	Thermo Fisher Scientific
**NKp30**	CD337	PE	P30-15	Biolegend
**SLAMF6**	NTB-A/CD352	PE	NT-7	Biolegend
**Granzyme B**	Granzyme B	PE	GB11	Thermo Fisher Scientific
**HLA-DR**	HLA-DR	eFluor780	LN3	Thermo Fisher Scientific
**Perforin**	Perforin	PE	dG9	Thermo Fisher Scientific
**CD45RA**	CD45RA	APC, Brilliant Violet 421	HI100	Biolegend
**AHR**	AHR	PE	FF3399	Thermo Fisher Scientific
**AIOLOS**	IKZF3	PE	16D9C97	Biolegend
**CBFB**	CBFB	unconjugated	JG39-35	Thermo Fisher Scientific
**Donkey Anti-Rabbit**	Donkey Anti-Rabbit	PE	Poly4064	Biolegend
**EOMES**	EOMES	PE, eFluor660	WD1928	Thermo Fisher Scientific
**GATA2**	GATA2	PE	W17242A	Biolegend
**HELIOS**	IKZF2	APC	22F6	Biolegend
**HOBIT**	ZNF683	Alexa Fluor 647	Sanquin-Hobit/1	BD Biosciences
**ID2**	ID2	APC	ILCID2	Thermo Fisher Scientific
**IFN-γ**	IFN-γ	eFluor 660	4S.B3	Thermo Fisher Scientific
**IKZF2**	HELIOS	APC	22F6	Biolegend

**IRF8**	IRF8	APC	V3GYWCH	Thermo Fisher Scientific
**NFIL3**	E4BP4	PE	MABA223	Thermo Fisher Scientific
**PRDM1**	Blimp-1	PE	6D3	BD Biosciences
**RUNX2**	AML3, CBFA1	PE	D1L7F	Cell Signalling Technologies
**RUNX3**	AML42 CBFA3	PE	R3-5G4	BD Biosciences
**TBET**	TBET	PE	4B10	Thermo Fisher Scientific
**TNF-α**	TNF-α	PECy7	Mab11	Thermo Fisher Scientific
**TOX**	TOX	PE	TXRX10	Thermo Fisher Scientific

Allophycocyanin (APC), Peridinin Chlorophyll Protein-Cyanin5.5 (PerCP-Cy5.5), Phycoerythrin (PE), Phycoerythrin-Cyanin7 (PECy7)

2 Supplementary Table 2. Utilized qPCR primers

Gene	Sense	Sequence
IRF2	forward	5'-AAGCACACTGAGAGGGCAC-3'
	reverse	5'-CATCCCACCCATGTCTAGCC-3'
IRF2 (flanking shRNA binding sites)	forward	5'-CGATGAAGAGAGTGCCGAG-3'
	reverse	5'-TTGAAAAGGGGGCCAGGAG-3'
BTG3	forward	5'-ATGAAATTGCTGCCGTTGTCT-3'
	reverse	5'-GCCTGTCCTTTCGATGGTTTT-3'
ZWILCH	forward	5'-TCCCTCCACTCTCTTCAACTGC-3'
	reverse	5'-CCAGTCCTCAAACCATCAGCCA-3'
CLSPN	forward	5'-TGAAAACTCAGGCAAGCAGCCTA-3'
	reverse	5'-GGCTGATAGGATGGAATCGTGG-3'
GAPDH	forward	5'-TCCTCTGACTTCAACAGCGACA-3'
	reverse	5'-GTGGTCGTTGAGGGCAATG-3'
TBP	forward	5'-CACGAACCACGGCACTGATT-3'
	reverse	5'-TTTTCTTGCTGCCAGTCTGGAC-3'
YWHAZ	forward	5'-ACTTTTGGTACATTGTGGCTTCAA-3'
	reverse	5'-CCGCCAGGACAAACCAGTAT-3'

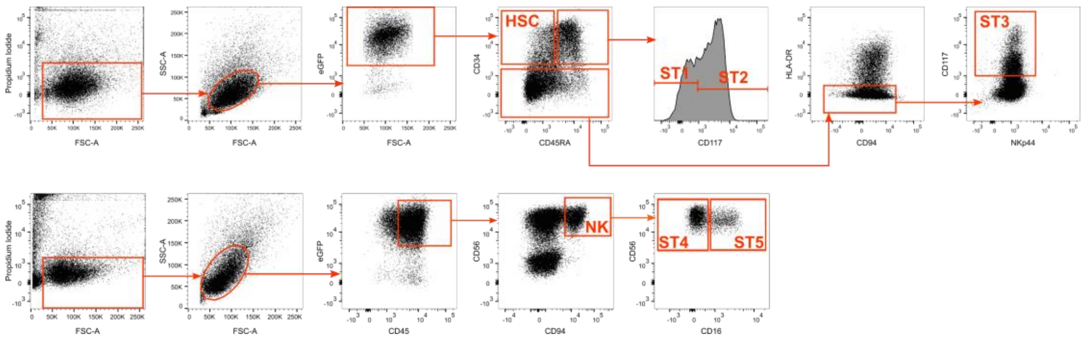

Supplementary Figure 1. Gating strategy of NK cell developmental stages in differentiation cultures. Flow cytometric gating strategy of NK cell developmental stages, including HSC (CD34$^+$CD45RA$^-$), stage 1 (CD34$^+$CD45RA$^+$CD117$^-$), stage 2 (CD34$^+$CD45RA$^+$CD117$^+$), stage 3 (CD34$^-$CD94$^-$CD117$^+$HLA-DR$^-$NKp44$^-$), stage 4 (CD45$^+$CD56$^+$CD94$^+$CD16$^-$) and stage 5 (CD45$^+$CD56$^+$CD94$^+$CD16$^+$).

Supplementary Table 3. Differentially expressed genes in d3 HSC and d7 ST3 cells in the gene ontology (GO) positive and negative regulation of mitotic cell cycle pathways. Genes containing an IRF2-binding motif are marked as bold.

**HSC**				**ST3**			
GO positive regulation of mitotic cell cycle		GO negative regulation of mitotic cell cycle		GO positive regulation of mitotic cell cycle		GO negative regulation of mitotic cell cycle	
**DOWN (4)**	**UP (4)**	**DOWN (12)**	**UP (4)**	**DOWN (6)**	**UP (7)**	**DOWN (15)**	**UP (14)**
CYP1A1	*RAB11A*	***TAOK3***	*INHBA*	***DYNLT3***	*DTL*	*CTDSP2*	*BRCA1*
CCND2	*NSMCE2*	*PTEN*	*BTG2*	*MEIS2*	*CDC6*	***TAOK3***	***CLSPN***
CDK4	*HES1*	*CTDSP1*	*BTG3*	***ANXA1***	*ANKRD17*	*CCL2*	*ZWILCH*
CCNB1	*EIF4E*	*TOPBP1*	*SDE2*	*PBX1*	*ANAPC5*	*ABRAXAS1*	*ZWINT*
		HMGA2		*PRKCA*	*AIF1*	*PTEN*	*DTL*
		BLM		***PAFAH1B1***	*BRCA2*	***CDKN1C***	*CDC6*
		CDK5RAP2			*EIF4E*	***FHL1***	*TPR*
		ZFP36L2				***CHMP4C***	*PRKDC*
		ORC1				***CDKN1A***	*BABAM1*
		CCNB1				***ZFP36L1***	*DONSON*
		NACC2				*NABP1*	*NDC80*
		FHL1				***CCNG1***	*MSH2*
						BTG2	***CDK5RAP2***
						BTN2A2	*MAD2L1*
						CRLF3	
